# Administration of Traditional Chinese Blood Circulation Activating Drugs for Microvascular Complications in Patients with Type 2 Diabetes Mellitus

**DOI:** 10.1155/2016/1081657

**Published:** 2016-10-18

**Authors:** Lisha He, Han Wang, Chengjuan Gu, Xinhui He, Linhua Zhao, Xiaolin Tong

**Affiliations:** ^1^Guang'anmen Hospital, China Academy of Chinese Medical Sciences, Beijing 100053, China; ^2^Beijing University of Chinese Medicine, Beijing 100029, China

## Abstract

Traditional Chinese medicine (TCM) is an important complementary strategy for treating diabetes mellitus (DM) in China. Traditional Chinese blood circulation activating drugs are intended to guide an overall approach to the prevention and treatment of microvascular complications of DM. The core mechanism is related to the protection of the vascular endothelium and the basement membrane. Here, we reviewed the scientific evidence underpinning the use of blood circulation activating drugs to prevent and treat DM-induced microvascular complications, including diabetic nephropathy (DN), diabetic peripheral neuropathy (DPN), and diabetic retinopathy (DR). Furthermore, we summarized the effects and mechanism of TCM on improving blood rheology, inhibiting aggregation of platelet, forming advanced glycation end products (AGEs), regulating oxidative stress, reducing blood fat, and improving lipid metabolism. The paper provides a new theoretical basis for the clinical practice of TCM in the prevention and treatment of DM and its microvascular complications.

## 1. Introduction

Diabetes mellitus (DM) is a complex, chronic, and common disease. According to the International Diabetes Federation (IDF), approximately 415 million people suffer from DM worldwide, and the number is predicted to increase to nearly 642.6 million by 2040 [1]. Diabetes is prevalent in China, with 109.6 million people afflicted with the disease, ranking it first in the world. Diabetes-related medical expenses in China were $51 billion in 2015, which is a huge burden on the country's health care system.

Diabetic microvascular complications commonly occur with DM, including diabetic nephropathy (DN), diabetic peripheral neuropathy (DPN), and diabetic retinopathy (DR) [2]. Due to the lack of early diagnostic markers, diabetic microvascular complications come to attention after the symptoms appear [3], and the prevention and the targeted therapeutic intervention of Western medicine is very limited [4, 5]. The multifactorial risk-reduction strategies of diabetic microvascular complications include addressing blood sugar and lipid control, weight management, and healthy lifestyle changes [6–8]. Nevertheless, more than two-thirds of the patients still do not meet the targets for glycemic goal of HbA1c < 7.0% in China [9], which can lead to the increased risk of microvascular complications.

Approximately 20–40% of patients with type 2 diabetes (T2DM) suffer from diabetic nephropathy, which is the leading cause of end-stage renal disease [10, 11]. ACE inhibitors [12] and ARBs [13, 14] have demonstrated the ability to protect renal function of DN but are not enough to retard the progression, and they are not recommended for the primary prevention of DN in patients who have normal blood pressure and normal UACR. DR is another complication that is the leading cause of vision loss [15]. Laser photocoagulation therapy [16, 17] and antivascular endothelial growth factor (VEGF) therapy [18, 19] are indicated for diabetic macular edema. Both are beneficial in reducing the risk of further visual loss but generally not beneficial in reversing already diminished acuity. DPN is an independent risk factor for cardiovascular mortality [20, 21]. Neuropathic pain is a typical clinical symptom, which can reduce the quality of life and limit patients' mobility [22], and drugs have only been approved specifically for the relief the neuropathic pain [23, 24], not to reverse neuronal loss [25, 26].

TCM has a 2,000-year history in treating DM and its complications in patients in China. In recent years, Chinese medicine has made a breakthrough in the treatment theory of diabetes [27]; significant evidence exists which supports the thought that TCM interventions can independently treat diabetes [28–30], and numerous studies have demonstrated that traditional Chinese blood circulation activating drugs have obvious advantages in the prevention and treatment of diabetic microvascular complications [31–33]. However, more research is needed to explore their mechanisms. In this review, we introduced the relevant mechanisms of the traditional Chinese blood circulation activating drugs on the treatment of diabetic microvascular complications based on the clinical and experimental studies.

## 2. Blood Stasis Is the Common Pathological Basis of Diabetic Microvascular Complications

Blood stasis is one of the important pathological concepts in traditional Chinese medicine, which is generally a significant pathological product due to the disorder of blood circulation [34]. The leading causes of blood stasis include Qi deficiency, Qi stagnation, and cold retention [35]. The blood stasis syndrome is characterized by symptoms such as pain in a fixed position, nyctalgia, dark-purple coloring of the tongue or face, infraorbital darkness, sublingual varicosis, blood spots under the skin or tongue, or an astringent pulse that can manifest [36]. Patients with microvascular complications often have the same symptoms and signs of blood stasis.

Researchers reported that high glucose environment causes the blood circulation obstacle and leads to blood stasis, and blood stasis is the core pathogenesis of microvascular complications [37, 38]. Promoting blood circulation to remove blood stasis is the main treatment principle [39]. As many complications of diabetes usually take years to develop before becoming clinically apparent, experts believe that the blood stasis not only is in the end stage but also exists throughout the course of the disease [40]. Zhou et al. [41] proposed the Luo disease (络病) theory and promoted the use of blood circulation activating drugs together with hypoglycemic agents at the beginning and through the duration of the treatment [42].

Blood stasis syndrome is similar to Western medicine's disturbance of microcirculation in which blood viscosity increases and causes blood coagulation. These pathological changes in the occurrence and development of the diabetes process have been confirmed [43]. Therefore, modern research provides a basis for the treatment of microvascular complications with the method of promoting blood circulation and removing blood stasis.

## 3. Blood Circulation Activating Drugs for Microvascular Complications

The representative herbs and their application in microvascular complications, as well as proposed mechanisms of action and efficacy for treating diabetes, are described in Table 1.

## 4. Evidence of Traditional Chinese Blood Circulation Activating Drugs in the Treatment of Diabetic Microvascular Complications

### 4.1. Diabetic Nephropathy

Qi deficiency and blood stasis are the core pathogenesis of DN; invigorating Qi and promoting blood circulation herbs can reduce the diabetic nephropathy proteinuria, protect renal function, and delay the progress of DN [44]. Li et al. [45] carried out a multicenter randomized, double-blind, placebo-controlled trial and reported that, based on conventional treatment with ACEIs or ARBs, Tangshen Formula (including Notoginseng Radix) provided additional benefits in decreasing proteinuria and improving eGFR in DN patients with macroalbuminuria. Tian's group retrospectively analyzed clinical outpatient medical records of DN and reported that Didang Formula (including leeches and* S. miltiorrhiza*) showed beneficial effects on the reduction of proteinuria and delaying the progression of DN [46].

Multiple clinical research teams [47–49] confirmed that, in the routine treatment with Western medicine, application of the kidney nourishing and blood activating formula (including Notoginseng Radix,* S. miltiorrhiza*) combined with enalapril [50] or irbesartan [51] to treat DN patients, had better efficacy in controlling blood glucose, blood lipid, urine albumin excretion rate (UAER), serum creatinine (SCr), and 24-hour urinary protein quantity compared with the simple Western medicine group.

### 4.2. Diabetic Retinopathy

DR is caused by microangiopathy and capillary closure, which results in the breakdown of the blood-retinal barrier with retinal hemorrhage, exudate and edema formation, and macular edema [52]. The improvement of ischemia and the protection of normal microvasculature in retinal tissue by blood circulation activating traditional Chinese drugs, which can save the visual acuity and inhibit progression of DR patients, have been confirmed [53].

Lian et al. [54] reported that, compared with the placebo, Compound Danshen Dripping Pill (CDDP, consisting of* Salvia miltiorrhiza*,* Panax pseudoginseng* var.* notoginseng*, and* Dryobalanops aromatica* Gaertn.f.) has definite efficacy in fluorescence fundus angiography (FFA) and funduscopic examination parameters for treating nonproliferative diabetic retinopathy (NPDR). Luo et al. [55] reported that CDDP has similar improvement and safety profiles to calcium dobesilate for NPDR in 57 type 2 diabetes cases. In future DR treatments, CDDP may function as the auxiliary drug. Luo et al. [56] reported that Qiming Granule (including typhae and leeches) can reduce the retinal blood circulation time (AVCT) and arm-to-retinal circulation time (ARCT), which may alleviate retinal hypoxia and ischemia by increasing retinal blood flow and improving blood circulation.

### 4.3. Diabetic Peripheral Neuropathy

The most common symptoms of DPN are induced by the extremity blood microcirculation disorder, including pain, dysesthesias (unpleasant abnormal sensations of burning and tingling), and numbness. The main treatment principle of TCM is supplementing Qi and activating blood circulation; a number of interventions have been demonstrated to reduce the risk and slow the progression of DPN.

Feng et al. [57] reported a successful treatment case of DPN pain using a high dose of the traditional Chinese medicine, Huangqi Guizhi Wuwu Tang (including astragalus, cassia twig, and spatholobi) in combination with aconitum. Qi and Yu [58] reported that the use of Mudan granule (containing* Ligusticum* chuanxiong,* Salvia miltiorrhiza*, hematoxylin, suberect spatholobus stem, and Notoginseng Radix) combined with Methylcobalamin can improve nerve conduction velocity and reduce the pain of PDPN. A clinical study found that Danggui Sini Tang (including* Angelica*) can help improve the conduction velocity of the common peroneal nerve in DPN treatment [59]. Gao et al. [60] observed that Tangluoning (TLN) can improve the nerve conductive velocity and clinical symptoms in DPN patients and the therapeutic mechanism may be induced by antioxidative effect.

## 5. Mechanisms of Blood Circulation Activating Drugs Used to Treat Diabetic Microvascular Complications

**Figure 1:**
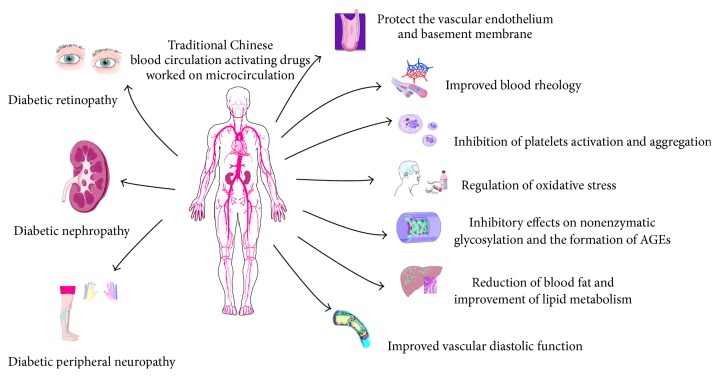
Effects and molecular mechanisms of traditional Chinese blood circulation activating drugs on diabetic microvascular complications treatment.

The mechanisms of the pathological process of DM-induced microvascular complications are complex. It is generally believed that hyperglycemia-induced oxidative stress is the important common mechanism, thus leading to further activation of the polyol pathway, nonenzymatic glycosylation, protein kinase C (PKC), and the hexosamine pathway, which eventually lead to chronic microvascular complications. In addition, microvascular complications are associated with vascular endothelial dysfunction, blood coagulation, and abnormalities of fibrinolytic system activity. Blood circulation activating drugs could be effective in these fields as natural supplementary medicines to complex, multilink, and multitarget drugs (Figure 1).

### 5.1. Protecting the Vascular Endothelium and Basement Membrane

The normal microvessel wall is based on the basement membrane. High glucose induces injury to vascular endothelial cells, which leads to progressive thickening of the basement membrane and obstruction of the involved microvascular, thus resulting in tissue hypoxia and increased lesions [61]. Patients with more vascular endothelial cells and a higher level of plasma endothelin have more incidences of getting microvascular diseases. Blood circulation activating drugs can protect the function of vascular endothelial cells and the basement membrane so as to prevent and treat diabetic microvascular complications [62].

Some researchers used the diabetic injury of human umbilical vein endothelial cells (HUVECs) as a model and found that Jiawei Taohe Chengqi Decoction (including peach kernel) through the regulation of dimensional protein plasminogen activator inhibitor-1 (PAI-1) improved vascular endothelial function [72]. Xuefu Zhuyu Decoction (including* Carthamus tinctorius* L.,* Angelica sinensis*, peach kernel, and* Ligusticum wallichii*) can protect the endothelial function by improving the DPN's ischemia and hypoxia state and regulating the microcirculation disturbance [63]. CDDP may protect vascular endothelial cells through its ability to eliminate free radicals [73]. Buyang Huanwu Decoction (including* Angelica sinensis*, Radix paeoniae rubra,* Ligusticum wallichii*,* Carthamus tinctorius* L., and peach kernel) can improve diabetic endothelial dysfunction and slow vascular cell proliferation at the systematic and local ocular level and improve microcirculation and the ability of tissue to be oxygen resistant [74, 75].

### 5.2. Improved Blood Rheology

Microvascular hemodynamic changes are an early sign of diabetic microvascular disease. In the early stage of diabetes, there is an increase in blood flow and high perfusion in the kidney, retina, and other tissues [76]. Due to the increase in microvascular permeability, the protein can seep out and deposit in the vascular wall causing microvascular thrombosis and occlusion, which results in serious overall circulation damage.

*Salvia miltiorrhiza* can protect STZ-induced diabetes rats from developing diabetic nephropathy, by suppressing the overexpressions of TGF-*β*1, CTGF, PAI-1, and FN in the renal cortex [65].* S. miltiorrhiza* also improves ischemia reperfusion and microcirculation [77–79]. CDDP may improve blood flow of the local tissue to prevent thrombosis. Leeches can improve renal hyperfiltration and high perfusion of DN rats in the first stage; the mechanism may work through the downregulation of endothelial endothelin-1 (ET-1) expression [80]. Huang et al. [81] used* Ginkgo biloba* extract (EGb761) to treat T2DM patients for 3 months; the results showed that diabetic patients' blood coagulation degree was obviously decreased, erythrocyte binding capacity was enhanced, and retinal capillary blood flow velocity was significantly accelerated.

### 5.3. Inhibition of Platelets Activation and Aggregation

Red blood cell aggregation increased in patients with diabetes, showing a high coagulation state and abnormal release of oxygen. High platelet adhesion, abnormal anticoagulation, and blood viscosity increased, resulting in luminal stenosis and microcirculation, which work together to promote the occurrence of microvascular lesions [82].

Studies have shown that blood circulation activating drugs such as* Ligusticum* chuanxiong and Typhae Pollen [83] and blood circulation activating formulas, including Buyang Huangwu Formula [84], Danggui Sini Formula [85], Huangqi Guizhi Formula [86], and Danhong injection [87], which can reduce blood viscosity, improve the high coagulation and high viscosity state. These prescriptions showed the effect of invigorating blood and hemostasis by ameliorating the abnormal hemorheological parameters and improving microcirculation, promoting oxygen and energy metabolism, and relieving clinical symptoms.

### 5.4. Regulation of Oxidative Stress

Oxidative stress is an imbalance between oxidation and antioxidation, an increase in ROS and oxygen free radicals* in vivo*, which is considered to be an important factor leading to aging and disease [88, 89]. Ceriello and Motz [90] proposed that oxidative stress is the common basis of insulin resistance, diabetes, and cardiovascular disease. According to the common mechanism of diabetic complications, it suggests that the damage to endothelial cells by oxidative stress is the common upstream mechanism of hyperglycemia-related vascular diseases [71].

Researches revealed that* Panax notoginseng* [68, 91], curcumin [69],* S. miltiorrhiza* [92], Tongluo Formula [93] (including ginseng, leech, scorpion, and centipede), Huangqi Guizhi Formula [94], Danzhi Jiangtang Capsule (including leech) [95, 96], and Honghua injection [97] have antioxidative effects on the treatment of diabetic vascular complications. The main mechanisms reduce malondialdehyde content and increase superoxide dismutase activities to protect the vascular endothelial cells [98]. These antioxidative effects improve microcirculation disturbance, thereby increasing blood flow to remove blood stasis [99]. Antioxidant therapy has become another new way to prevent and cure diabetes mellitus and its complications [100].

### 5.5. Inhibitory Effects on Nonenzymatic Glycosylation and the Formation of Advanced Glycation End Products

Sustained hyperglycemia could cause a variety of nonenzymatic glycation of proteins and ultimately form advanced glycation end products (AGEs). The AGEs could bind to the specific receptors of mesangial cells, endothelial cells, and so forth via affecting the extracellular matrix, changing the intracellular signaling conduction, and then altering the structure and function of proteins in targeted organizations. Tissue deposition of AGEs is the main characteristic of diabetic microvascular complications.

Studies showed that* Salvia miltiorrhiza*, leech, Rhizoma Chuanxiong, Radix notoginseng, and so forth can block the formation of AGEs and lead to the inhibition of the activation of pathways conducted by RAGE, thereby inhibiting activation of pathways, including ERK1/2, p38, JAK stat, and PI3K [101]. These herb medicines can also downregulate the expression of ROS by inhibiting the activation of NADH/NADPH pathway, which result in the downregulation of vascular injury proteins and cytokines [102]. According to the abovementioned multitarget mechanisms, the principle of promoting blood circulation by removing blood stasis became an important tool in the treatment of diabetic vascular complications.

### 5.6. Reduction of Blood Fat and Improvement of Lipid Metabolism

Lipid toxicity is an independent risk factor for the onset of T2DM [103] and lipid metabolic disorder plays an important role in diabetic microvascular complications [104]. Elevated free fatty acid (FFA) levels could lead to the damage of blood vessel endothelium [105, 106] and also change the permeability basement membrane in endothelial cells, which result in the thickness of vascular intima [107]. Patients with T2DM are sensitive to fatty acids metabolism and lipid peroxidation, which leads to the increased formation of free radicals and lipid peroxide and the damage of the normal structure of the subcellular membrane in the cell nucleus.

Blood circulation activating drugs showed an antihyperlipidemic effect on lowering the levels of total cholesterol (CHO), triglyceride (TG), and FFA, improving the secretion function of *β* cells, which has been observed in both animals and humans [108, 109]. Clinical studies found that* Ginkgo biloba* leaves extract tablets can regulate blood lipid metabolism and reduce lipid toxicity in patients with early T2DM [110]. Mudan granules can improve blood glucose and blood lipids and reduce blood viscosity of DNP patients, which improves nerve conduction velocity and oxidative stress [111].

### 5.7. Improved Vascular Diastolic Function

Impaired endothelium dependent vascular relaxation (EDVR) plays an important role in the development of diabetic microvascular diseases. T2DM and other metabolic abnormalities can enhance oxidative stress, induce nitric oxide inactivation, which results in reduced endothelial NO production, and release a large number of oxygen free radicals and prostaglandin resulting in vascular diastolic dysfunction [112].

Studies showed that ligustrazine could improve the EDVR of diabetic rats and rhubarb can reduce plasma endothelin-1 (ET-1) and increase the role of NO in diabetic rats, which can protect the EDVR, and inhibit ICAM-1 and VCAM-1 expression in the thoracic aorta [113].

## 6. Summary 

The treatment of DM and its complications mainly depends on Western drugs, but sometimes the clinical efficacy is far from satisfactory because of the failure to delay the progression of diabetic complications. TCM plays an important role in the treatment of DM and its complications in China via diverse ways. We confirm that the blood circulation activating drugs, both compound and formula, have a certain effect on the prevention and the treatment of DM-induced microvascular complications. More specifically, TCM herbs influenced not only blood glucose but also the microvascular environment and some signal pathways.

Further analysis of the blood circulation activating drugs* Salvia miltiorrhiza*,* P. notoginseng*, Typhae Pollen,* Angelica sinensis*,* Ligusticum wallichii*,* Carthamus tinctorius* L., Caulis Spatholobi, leech, and so forth shows that certain kinds of TCM compounds, which have different roles in treating and preventing the microvascular complications, could have multiple effects in the treatment of the disease. Treatment based on syndrome differentiation (ZHENG) is a basic rule of TCM treatment [114]. According to the principle, the main syndrome patterns at the end stage of diabetic microvascular complications can be divided into Qi deficiency with blood stasis (气虚血瘀), Yin deficiency with blood stasis due to cold (阴*虚寒凝*血瘀), and phlegm with blood stasis (痰瘀互结). These herbs need to be cataloged and cooperated with other drugs according to patients' TCM ZHENGs. The tonified medicines Radix Codonopsis, Radix Ginseng, and Astragali Preparata are used to treat the Qi deficiency ZHENG, while Radix Rehmanniae and Radix Trichosanthis relieve the Yin deficiency ZHENG.

Significant evidences exist that support a range of TCM interventions to improve the diabetes outcomes. Blood circulation activating drugs are critical to prevent acute complications and reduce the risk of long-term complications. However, most of the evidence-based researches were carried out in Asian countries, and further studies should be performed in other continents so as to account for the population-dependent variation of efficacy and confirm the universality of the benefits of these drugs. Above all, it is worth a further study to figure out the relationship and biochemical mechanism of different catalogs of TCM herbs in treating diabetic complications. With the rapid development of technology, TCM will play a more important role in complementary and alternative therapies for DM.

## Figures and Tables

**Table 1:** The representative blood circulation activating herbs for microvascular complications.

Pin Yin name	Latin (scientific) or English name	Function
Danshen	*Salvia miltiorrhiza*	Promote blood flow and hemostasis, antioxidative effect [61–63]
Sanqi	*P. notoginseng*	Invigorate blood and hemostasis, antioxidative effect [64]
Puhuang	Typhae Pollen	Inhibition activation and aggregate platelets [65]
Danggui	*Angelica sinensis*	Protect vascular endothelial function [59, 60]
Chuanxiong	*Ligusticum wallichii*	Protect vascular endothelium [57, 59, 60]
Honghua	*Carthamus tinctorius *L.	Invigorate blood and hemostasis, antioxidative effect [57, 66]
Jixueteng	*Caulis Spatholobi*	Inhibit activation and aggregate platelets [67]
Taoren	Peach kernel	Inhibit aggregate of platelets and invigorate blood and hemostasis [42, 58, 59]
Chishao	Radix paeoniae rubra	Lower lipids and decrease oxygen free radicals [61, 62]
Quanxie	Scorpion	Improve blood rheology [68, 69]
Shuizhi	Leech	Lower lipids and decrease oxygen free radicals [66, 69]
Wugong	Centipede	Improve blood rheology [70, 71]
